# PARTNERING TO DEVELOP COUNTY-LEVEL DATA PROFILES

**DOI:** 10.1093/geroni/igac059.2147

**Published:** 2022-12-20

**Authors:** Karon Phillips, Megan Wolfe

**Affiliations:** Trust for America's Health, Silver Spring, Maryland, United States; Trust for America's Health, Washington, District of Columbia, United States

## Abstract

Building robust data systems that include information on the health of older adults is crucial to developing programs and services that meet their health and social needs. Organizations and agencies can use this information to target resources and identify community partners, as well as using data to support grant applications. But there are challenges to building such systems, even within the public health system, due to lack of expertise, funding, and access to underlying data sources. Through its Age-Friendly Public Health Systems initiative, Trust for America’s Health’s has worked directly with several state public health departments to explore opportunities to develop and expand their county-level data profiles on older adults. This session will highlight the unique collaborations and partnerships between departments of health and other institutions, including academia, to develop data profiles. Through these innovative partnerships, departments of health have been able to develop dynamic data profiles that they share with aging services providers to discuss strategies to support their older adults in their communities. Best practices and lessons learned through the profile development process will also be discussed.

